# Patient with Mutation in the Matrix Metalloproteinase 2 (MMP2) Gene - A Case Report and Review of the Literature

**DOI:** 10.4274/Jcrpe.1166

**Published:** 2014-03-05

**Authors:** Alka V. Ekbote, Sumita Danda, Andreas Zankl, Kausik Mandal, Tina Maguire, Kobus Ungerer

**Affiliations:** 1 Christian Medical College, Clinical Genetics Unit, Vellore, India; 2 University of Queensland, Bone Dysplasia Research Group, UQ Centre for Clinical Research, Herston, Australia; 3 University of Queensland, Department of Botany, Brisbane, Queensland, Australia; 4 Queensland Health Services, Royal Brisbane and Women Hospital, Herston, Australia

**Keywords:** Torg syndrome, MMP2, osteolysis

## Abstract

Torg and Winchester syndromes and patients reported by Al-AqeelSawairi as well as nodulosis-arthropathy-osteolysis (NAO) patients, patients with multicentric NAO share autosomal recessive inheritance. The common presenting symptomatology includes progressive osteolysis chiefly affecting the carpal, tarsal and interphalangeal joints. Here, we report a patient with Torg syndrome. Torg syndrome is caused by homozygous or compound heterozygous mutations in the matrix metalloproteinase 2 (MMP2) gene. MMP2 codes for a gelatinase that cleaves type IV collagen, a major component of basement membrane. The clinical presentation of our patient included moderate osteolysis of the small joints of the hands and knees, hirsutism, nodulosis sparing the palms and soles, corneal opacities and mild facial dysmorphism without gum hypertrophy. Genetic analysis showed that the patient was homozygous for a novel base variant c538 G>A (p.D180N) in the MMP2 gene. Both parents were carriers of the same mutated variant. Our patient had some previously unreported endocrine manifestations such as premature thelarche and elevated follicle-stimulating hormone levels.

## INTRODUCTION

Autosomal-recessive genetic osteolysis disorders include infantile systemic hyalinosis, mandibulo-acral dysplasia type A and B, Winchester and Torg syndromes [including nodulosis-arthropathy-osteolysis (NAO) syndrome]. Winchester syndrome was originally described in 1969 as a mucopolysaccharidosis with skeletal deformities (1). The Torg syndrome was first defined in 1969 (2). Al Mayouf et al (3) described a similar entity with NAO in ten unrelated members of six Saudi Arabian families. Martignetti et al (4) in 2001 reported that mutations in matrix metalloproteinase 2 (MMP2) gene are pathogenic for NAO syndrome. Zankl et al (5) proposed in 2005 that the above-mentioned clinical entities are allelic and are based on presence of similar mutations and hence be named as a single entity (as Torg-Winchester syndrome). A study by Evans et al (6) in 2012 redefined the molecular basis of Winchester syndrome and proposed that it should be accepted to be a single gene disorder distinct from both Torg and NAO syndromes. According to these authors, the clinical presentation of this syndrome is diverse and includes resorption of digits, joint affection including contractures, subcutaneous nodules, distinctive facial features, growth deficiency and endocrine abnormalities. In this syndrome, serum and immune inflammatory bone markers are distinctively absent.

We present here a patient with a clinical phenotype resembling the Torg syndrome. Molecular analysis of the MMP2 gene revealed a novel base variant in the gene MMP2 c538 G>A (p.D180N). We discuss our patient in comparison to previous reports on patients with mutation in the same codon as well as patients originally described as Winchester syndrome. We also discuss novel endocrine manifestations of the disease that include premature thelarche and increased levels of follicle-stimulating hormone (FSH).

## CASE REPORT

A female child aged 3 years and 5 months presented with progressive swelling and deformity of her small and large joints. The symptoms were first noted 6 months prior to this visit. She also had poor weight gain since age one year. The interphalangeal joints as well as the left and right metacarpal joints were reported as the initially involved joints. The symptoms gradually progressed to involve the wrist, elbow and knee joints, eventually leading to limitation of the movements of the involved joints.

The patient was born by normal vaginal delivery to a non-consanguineous couple. Antenatal period was uneventful. She was noted to have bilateral congenital talipes equinovarus deformity at birth. The deformity was corrected using a corrective plaster cast. Her developmental milestones were normal for age, but she had regression of motor milestones with involvement of lower limb joints. She has one younger brother who is healthy to date.

**Figure 1:**
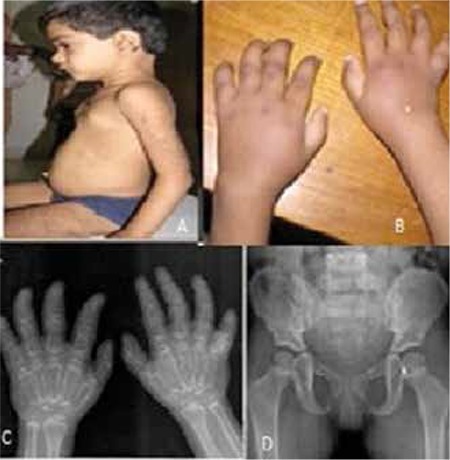
A) Hirsutism on the elbows and back, a hairy nevus over the pinna, premature breast enlargement B) Hyperpigmented shiny skin, excessive skinfolds (telescoping) near the little finger; osteolysis, swelling and widening of the wrist C) X-ray of the hand and wrist showing resorption of distal phalanges and loss of bone mineralization in the phalangeal and carpal bones. There is cortical thinning and expansion of metacarpal bones. Similar changes are visible at the wrist involving the distal ends of the radius and ulna D) X-ray of the pelvis showing a lesser degree of osteopenia

Anthropometric measurements included a weight of 8 kg [<5 standard deviations (SD)], height of 75.5 cm (<3 SD), head circumference of 44.6 cm (<3 SD) according to reference values for the Indian population. Physical examination revealed mildly coarse facial features, increased body hair over the back and extremities, a hairy nevus over the left ear and breast enlargement. Ophthalmologic, as well as ENT (ears, nose, throat) and dental findings were unremarkable. There was a swelling over the dorsum of the right hand and wrist and also a swelling and fixed flexion deformity of the left metacarpophalangeal joint and of both knees. Also, the patient displayed flexion deformity in all fingers at the distal interphalangeal joint. The skin of the hands and knuckles was hyperpigmented. Both elbow joints and the third and fourth fingers on the right side had subcutaneous nodules on the dorsum (Figure 1).

The X-rays showed loss of bone mineralization of the involved joints, decrease in joint space and resorption of phalanges. Cortical thinning and expansion of the phalangeal and metacarpal bones was a distinct finding. The rest of the skeleton showed a lesser degree of osteopenia. The immunological and hematological reports were normal except for a high erythrocyte sedimentation rate (20 mm at 1 hour). Phenotypic characteristics and biochemical values are depicted in Table 1 and Table 2.

Abdominal ultrasonography (US) was unremarkable except for a small-sized uterus (21.4 x 12.1 x 6.4 mm). The ovaries were slightly enlarged and measured as: right ovary 25.6 x 14.2 x 12.5 mm and left ovary 17.7 x 11.5 mm.

The patient was diagnosed to have Torg syndrome based on her history, physical examination and radiological findings.

**Molecular Analysis**

Written informed consent of the parents was taken as per the guidelines of the Institutional Ethics Committee. Qiamp mini kit was used to extract the DNA of the propositus, that of the parents and sibling. The sequencing of all exons of MMP2 gene was done using published methods and primers (courtesy of Queensland Health Pathology Services, Royal Brisbane and Women’s Hospital, Herston Australia) (5,7).

The results showed a novel base variant of MMP2:c.538G>A (p.D180N) in the homozygous state in the patient. The parents carried the variant in the heterozygous state. The sibling showed no mutation. We performed the in silico analysis using online tools [Polyphen2, SIFT (sorting intolerant from tolerant) and Pmut. Polyphen2 (score 0.989, sensitivity 0.72 and specificity 0.97) and SIFT (score 0, Median information content 2.7)] which predicted this variant as pathogenic.

**Figure 2:**
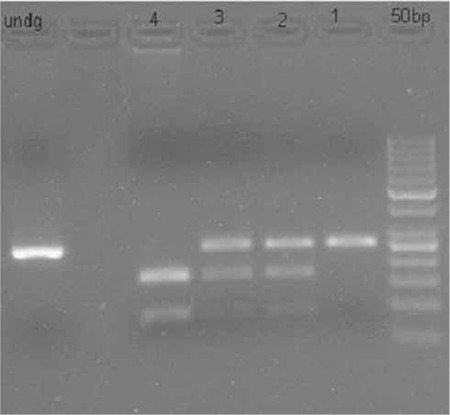
Restriction fragment length polymorphism with Btgz1 (from left to right). Lane 1: DNA marker 50 basepair; Lane 2: Proband; Lane 3, 4: Parents; Lane 5: Unaffected sibling; Lane 6: Blank; Lane 7: Undigested products. The Btgz1 does not cut the homozygous mutated allele (propositus). The parents are heterozygous for one mutant (undigested) and two bands representing wild type allele. The sibling had homozygous wild type allele

We tested this variant in 100 chromosomes by restriction fragment length polymorphism (RFLP) designed for the wild type allele using enzyme Btgz1. Homozygous wild state had 2 bands of 174 and 81 base pairs (bp). The heterozygous state shower three bands of 255bp, 174bp, 81bp and the mutated homozygous state did not cut and had one band at 255bp (Figure 2). None of the 100 chromosomes taken from the normal population carried the mutated variant either in heterozygous or homozygous state. The unaffected sibling showed a homozygous state for the wild variant.

## DISCUSSION

**Figure 3:**
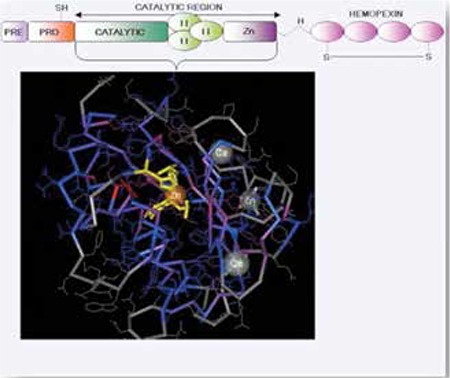
Domain structure of MMP-2 (Reproduction from Atlas of Hematology and Cytogenetics) This is a 3D-structure of the active site showing the area affected by mutation in the Yellow (calcium binding) site which is adjacent to the TIMP binding site. The site is a conserved region amongst the various MMPs of different species. Zinc is also an essential cofactor for the enzyme domain.Mutations in this domain can lead to destabilization of enzyme-cofactor complex

Mutations in the MMP2 gene are responsible in the pathogenesis of both Torg and NAO syndromes. This gene has 13 exons and encodes for transcript having 660 amino acids (http://www.ncbi.nlm.nih.gov/gene/4313). The protein is a gelatinase and is released as a pro-enzyme. The domain structure of the protein can be divided into five regions (Figure 3) (7,8). Mutations can be small deletions, splicing errors, or homozygous and compound heterozygous loss-of-function single-base changes (5,6,8).

MMPs, also referred to as matrixins, are zinc-dependent endoproteases known to play a role in the degradation of extracellular matrix (ECM). These are key molecules in the regulation of embryonic development, tissue remodeling, menstruation and reproduction. They also have a pathogenic role in arthritis and various cancers (9).

MMPs are also highly regulated post-transcription and post-translation factors. Tissue inhibitor of metalloproteinase type 2 (TIMP2) and MT-MMP1 (membrane type MMP, MMP14) together regulate the activity of MMP2. MMP14 is a membrane-bound protease playing a key role in maintenance of homeostasis during ECM degradation by forming a complex with TIMP-1, TIMP-2 and pro-MMP2. MMP14 also regulates the cleavage of proMMP2 to active MMP2. Recent research has also shown that expression of all these three proteins is co-regulated (9,10,11).

Our patient had a novel base change c538G>A in the MMP2 gene. This had led to alteration of the amino acid aspartic acid to asparagine at position 180 of the transcript (p.D180N). This amino acid resides in the conserved zinc-binding site of the catalytic domain. Cleavage or decreased neutralization by the above complex may lead to deregulated enzyme activity. Another mutation causing alteration in the same codon (D180N) has been reported before in 3 siblings presenting with Torg syndrome, further supporting the pathogenesis (12). Phenotypic comparison of our patient with these patients as well differences and similarities with the Winchester syndrome are presented in Table 1.

Phenotypic characteristics of our patient were similar to the family described by Temtamy et al (12) except for thelarche, growth retardation and elevated serum FSH levels. The severity and onset of disease are variable. Compared to the Winchester syndrome, phenotypic features are milder in Torg patients. Absence of severe facial coarsening, gum hypertrophy, corneal clouding, electrocardiography changes and severe vertebral and membranous as well as tubular bone change are a part of Winchester syndrome but are absent in Torg syndrome. Cardiac symptoms, nodules and vertebral involvement are also less severe in Torg cases.

The findings of increased body hair and hyperpigmentation in our patient were as described. Almost all the patients described with Torg phenotype in the literature had hyperpigmentation and hirsutism (2,3,5,13).

The severity and nature of joint involvement were comparable amongst patients presenting with the same mutation. Osteopenia and osteolysis of distal and middle phalangeal joints with telescoping of the skin was similar in all patients. Thinning and expansion of the cortices of proximal phalanges and metacarpals were present. There was bilateral restriction of movement of interphalangeal, metatarsophalangeal joints, wrists, elbows, feet and hips. The knees were involved to a variable extent. The shoulder joint involvement was observed in the female patient described by Temtamy et al (12) but was absent in our patient. Extensive changes in the lower tibial metaphyses, ankylosis and destruction of joints, spinal involvement with C1-C2 subluxation, widening of cranial sutures as well as ends of clavicles were only described in patients with Winchester syndrome. The Torg syndrome is less severe than the Winchester syndrome in terms of severity of osteolysis, neurological involvement and involvement of membranous bones (1,6,11,12,13). Neurological involvement is not observed in all patients described with this mutation (1,6).

Presentation of the patients described by Temtamy et al (12) is similar to our patient except growth retardation and lack of endocrine involvement. Growth retardation similar to that in our patient was present in the family described by Tuysuz et al (8). The evidence is lacking to assign growth retardation to this particular mutation. Phenotypic variability is seen even among the members of the same family in this syndrome.

Cardiac involvement has been previously described in the Torg syndrome. The link between MMP2 mutation and cardiac pathology was first described by Tuysuz et al (8). These authors reported three children from an extended consanguineous family who presented with congenital heart disease and who had mutations in the hemopexin domain. The authors argued that the MMP2 mutations when studied in the chick and mouse hearts blocked the epithelio-mesenchyme transition, migration and invasion of explanted mouse atrio-ventricular canal cells involved in the endocardial cushion formation necessary for adequate heart development.

Cheng et al (14) reported a positive correlation between severity and spontaneous closure of ventricular septal defects with the circulating levels of MMP2 and MMP9.

Our patient had no cardiac anomaly, neither structural nor conduction related. Temtamy et al (12) reported mitral valve prolapse in one of the siblings. Absence of cardiac disease in our patient as well as in other children of the same family again shows the phenotypic variability associated with this particular mutation (12). It is obvious that this mutation does not lead to severe cardiac disease.

Diabetes and thyroid anomalies are frequently-reported endocrine diseases in this syndrome. So far, these have not developed in our patient. Our patient had hirsutism and thelarche (SMR stage-2), but did not show any other sign of precocious puberty. Biochemical analysis revealed high FSH levels, but LH levels were not high. Estradiol, testosterone and DHEAS levels were all normal (Table 2). We believe that MMPs could be responsible for the high levels of FSH as well as for thelarche. At the end of the first year of follow-up, the patient was found to maintain the same status of hormones and thelarche. Ovaries on the follow-up ultrasound were large with small cysts. Further follow-up may be necessary to detect development of precocious puberty.

The role of MMPs in developing mammary glands during puberty has been well studied (15,16). Formation of the mammary gland occurs by two distinct mechanisms: a) terminal end bud formation (TEB) that determines the length of the mammary duct and 2) secondary branching that determines the formation of the branches of the ducts. MMP2 null glands are deficient in TEB and MMP3 null glands show lack of lateral branching. This lack of MMP2 inhibition might explain the thelarche noted in our patient (6,15,16).

Temtamy et al (12) reported polycystic ovaries and a high LH/FSH ratio in the female sibling, while hormone levels were normal otherwise. There was no mention of hormone levels in the younger brother.

Evidence supporting the role of increased FSH levels as a cause of stress is lacking. Published research on the relationship between high FSH levels and testes development was investigated in mouse models. The tissue inhibitor of metalloproteases type 1 and 2 (TIMP1-2) are regulated by the FSH released from Sertoli cells of developing testes in the mouse (17).

Portela et al (18) have shown that MMP2 and MMP9 play no role in the control of ovarian hormone levels. Evidence on the role of MMP in hormonal control is restricted to animal models, but it would be inadequate to altogether ignore these findings.

We also have found that nodules were present in our patient. According to Jeong et al (19), mutation in the catalytic domain of the MMP2 gene does not lead to nodule formation. Our patient is an antithesis to that hypothesis.

The treatment of Torg syndrome is supportive and includes management of the joint involvement in the departments of orthopedics, rehabilitation medicine and medical genetics. In an uncontrolled trial of cyclic pamidronate, a favorable effect was reported on bone pain and bone mineralization, especially pertaining to the vertebral bones. Others have reported no improvement with bisphosphonates (20). None of these studies have shown a decrease neither in the progression of the disease nor in alkaline phosphatase activity. Our patient had undergone a trial of both immunosuppressant drugs and pamidronate before presenting to our Unit, with no significant improvement. Genetic counseling plays an important role at every step of diagnosis and treatment. Molecular analysis of patients with genetic osteolytic syndromes is recommended for appropriate genetic counseling and also to aid in developing crucial molecules for targeted therapy.

**Acknowledgement**

We thank Mr. Ranjan (D.M.LT) for doing the laboratory analysis of the patient.

## Figures and Tables

**Table 1:**
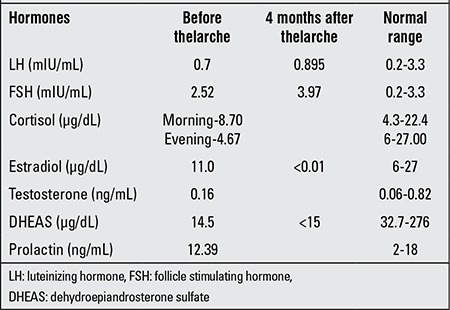
Hormonal evaluation of the patient before and after the onset of thelarche

**Table 2:**
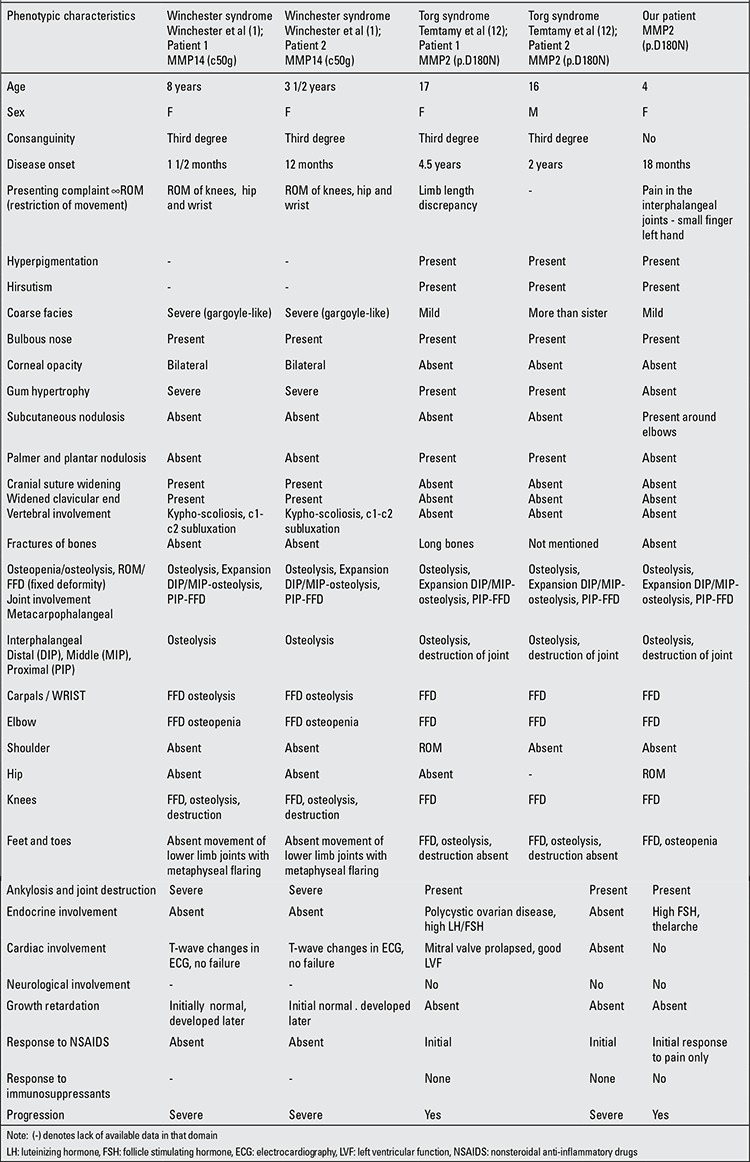
Comparison of the phenotypic characteristics of our patient with a patient with similar mutation reported by Temtamy et al ([ref:12]12[/ref]) and original cases presented by Winchester et al ([ref:1]1[/ref])
